# Determining behavioral intention and its predictors toward preconception care use among reproductive age women in Arba Minch town, Southern Ethiopia, 2022: a cross-sectional study based on the theory of planned behavior

**DOI:** 10.1186/s12884-024-06737-z

**Published:** 2024-08-23

**Authors:** Habtamu Alemu Tena, Kassahun Fikadu, Gebremariam Temesgen Birgoda, Abel Belete Cherkos, Tesfahun Simon Hadaro, Begetayinoral Kussia Lahole

**Affiliations:** Department of Midwifery, College of Medicine and Health Sciences, Arba Minch University, Arba Minch, Ethiopia

**Keywords:** Preconception care, Intention, Reproductive age women, Theory of planned behaviors

## Abstract

**Background:**

Preconception care is a highly effective, evidence-based intervention aimed at promoting the health of reproductive-age women and reducing adverse pregnancy-related outcomes. The Ethiopian Ministry of Health plans to integrate preconception care services into the country’s existing healthcare system. However, women’s preferences may be influenced by their values and customs. Therefore, this study used the theory of planned behavior to assess women’s intention toward preconception care use and its predictors among reproductive-age women in Arba Minch town, southern Ethiopia.

**Methods:**

A community-based cross-sectional study was conducted in Arba Minch town from May 1 to 30, 2022. A simple random sampling technique was employed to select 415 study participants for data collection. Data were collected through a face-to-face interview using a pretested, structured questionnaire. Epi Data version 4.6 and SPSS version 26 were used for the entry and analysis of data, respectively. Multiple linear regression was performed to identify independent predictors of intention to use preconception care. The standardized β-coefficient was used as a measure of association. A *P* value of less than 0.05 was used to declare statistical significance.

**Results:**

This study had 415 participants, giving a response rate of 98.3%. The mean age of the participants was 28.4 (SD 5.18). The mean intention to use preconception care was 21.43 (SD 2.47). Direct perceived behavioral control (β = 0.263, *p* < 0.001), direct attitude (β = 0.201, *p* = 0.001), direct subjective norm (β = 0.158, *p* = 0.006), and age (β=-0.115, *p* = 0.023) were significant predictors of women’s behavioral intention to use preconception care.

**Conclusion:**

The study identified perceived behavioral control as the strongest predictor, followed by attitude and subjective norms, influencing the intention to use preconception care. These findings underscore the importance of integrating these predictors into health intervention programs aimed at promoting the implementation of preconception care services.

## Introduction

Preconception care (PCC) is a crucial intervention recommended for individuals of reproductive age before pregnancy to optimize their health and promote favorable outcomes for both the mother and the baby. It involves addressing a set of biomedical, behavioral, and social health factors through interventions designed to identify and modify women’s health and pregnancy outcomes through prevention and management. Similarly, ‘inter-conception care’ refers to the provision of these interventions between two pregnancies [1, 2].

There is growing evidence that integrating PCC into the current health system can reduce maternal and child mortality and prevent unplanned and unwanted pregnancies, delivery complications, stillbirth, low birth weight, preterm labor, congenital anomalies, neonatal infections, stunting, and mother-to-child HIV/STI transmission [3, 4].

Despite the solid reasons for preconception care implementation, it is not integrated into the current maternal continuum of care in Ethiopia. Researchers identify several obstacles, including women’s lack of knowledge, inadequate education, experience of adverse pregnancy outcomes and ongoing health problems, as well as a shortage of trained health professionals and national policy guidelines [5–7]. Even though there are initiatives in terms of guideline development and integration of preconception care into health science curricula, the implementation of PCC remains a missed opportunity in Ethiopia’s contemporary healthcare system [8].

Furthermore, one of the reasons for the delay in implementing healthcare programs such as PCC is a lack of attention to research that takes into account social psychological models. Social psychological models are useful tools for understanding the factors that influence health behaviors and can help in designing effective interventions [9–11].

The Theory of Planned Behavior (TPB) is applicable to PCC, focusing on individual attitudes, perceived subjective norms, and perceived behavioral control [12]. According to this model, a person’s intention—the most critical determinant—is formed through a combination of their attitude, subjective norms, and perceived behavioral control [13–15]. Evidence on individuals’ intention to utilize preconception health services is crucial for predicting modifiable factors that influence the intrauterine environment and fetal development, such as diet, nutritional status, and lifestyle choices, which can be adjusted before conception [15].

However, little research has been conducted to evaluate predictors representing women’s intention to receive preconception care and its predictors. Furthermore, the evidence generated from such research should serve as a cornerstone in developing and implementing preconception care within the healthcare system. Therefore, this study aimed to assess the intention to use preconception care and its predictors among reproductive-age women in Arba Minch town from the perspective of the theory of planned behavior.

## Methods

### Study design, period, and settings

A community-based cross-sectional study was conducted from May 1st to May 30^th,^ 2022, at Arba Minch town, southern Ethiopia. Arba Minch town is located approximately 495 km south of Addis Ababa, the capital city of Ethiopia. The town has twelve kebeles (Ethiopia’s smallest administrative unit), with a total population of 123,446, of which 50.2% are females and 23.3% are of reproductive age. In the town, there are two health centers, one primary hospital, one general hospital, and more than 20 private health facilities. According to Arba Minch town’s Health Department 2014 EC report, the expected number of pregnancies and estimated deliveries for this year were approximately 4,271.

### Source participants

All women of reproductive age who lived in Arba Minch town were the source population.

### Study populations

Randomly selected reproductive-age women who meet the inclusion criteria.

### Sampling technique and sample size determination

Simple random sampling technique was used. Lists of households with women of reproductive age were obtained from health extension workers of respective kebeles. Samples were allocated to each kebeles proportionally to their size. Then, samples were drawn using computer-generated random numbers. Randomly selected households with eligible women were traced using their house numbers, and health extension workers were used as guidance. The Lottery method was used in households where more than one eligible woman was encountered. A single population mean formula was used to calculate the minimum sample size needed, with the assumption of a 95% confidence level, a 5% margin of error, and a 0.5 standard deviation of intention to use preconception care.

$${\rm{n = }}\frac{{{{\left( {\rm{Z}} \right)}^{\rm{2}}} * \left( {{\rm{SD}}} \right)\left( {{\rm{1 - SD}}} \right)}}{{{{\rm{d}}^{\rm{2}}}}}$$

where *n* = sample size.

SD = 0.5 of intention to use preconception care.

d = 5% margin of error.

Z = Value of standard normal distribution (Z-statistic) at 95% confidence level, which is 1.96.

Based on the above assumptions,

$${\rm{n = }}\frac{{{{\left( {{\rm{1}}{\rm{.96}}} \right)}^{\rm{2}}}{\rm{*}}\left( {{\rm{0}}{\rm{.5}}} \right)\left( {{\rm{1 - 0}}{\rm{.5}}} \right)}}{{{{\left( {{\rm{0}}{\rm{.05}}} \right)}^{\rm{2}}}}} \approx {\rm{384}}$$

For possible non-response during data collection time, 10% was added, which gave the final sample size of 422.

### Data collection procedure

The pretested interviewer-administered structured questionnaire was adapted from TPB constructs and different literature [16–18]. Before preparing the data collection tool, an elicitation study was performed by conducting in-depth interviews with 20 participants in the target groups to the locally available salient behavioral, normative, and control beliefs on preconception care use. Then, a structured questionnaire was prepared based on the TPB guidelines to collect data. The questionnaire has four sections: socio-demographic information, obstetrics and reproductive health characteristics, knowledge of preconception care, and the TPB. The first section collects demographic information, such as age, religion, marital status, employment, and education level. The second section covers the respondent’s obstetric and reproductive factors with five questions. The third section assesses the participants’ knowledge of preconception care with 12 items. The final section focuses on TPB, with 55 items separated into four subsets: intention, attitude, subjective norm, and perceived behavioral control. Four trained data collectors, Bachelor of Science degree holders in Midwifery, and one public health officer supervised the data collection process. Participants were engaged through local health authorities and selected using simple random sampling techniques. Interview locations were selected based on participants’ preferences, such as their homes, to ensure privacy and comfort. The average duration of the interviews ranged from 15 to 20 min.

### Data management and analysis

The completed questionnaire responses were entered into Epi-Data version 4.6 and exported to Statistical Package for Social Science (SPSS) version 26.0 for analysis. The recode command was used to recode negatively worded responses for the direct measures. Descriptive statistics were used and results were presented using tables. Pearson’s correlation analysis was conducted to determine the direction and relationship between the direct and indirect constructs of the theory of planned behavior and to confirm the validity of the indirect measures. The assumptions were checked for model fitness. All independent variables were first entered into simple linear regression, and those variables whose *P*-value was less than 0.25 were included in multiple linear regression. Adjusted R2 was used to determine the ability of explanatory variables to explain dependent variables. The standardized regression coefficient (β) was used to interpret the effect of predictors on the intention to use preconception care. A *P*-value of less than 0.05 was considered to indicate a significant association. Finally, the results were presented and summarized in text, tables, and numbers.

### Data quality control

The questionnaire was prepared in English and then translated into Amharic and back to English to check language consistency. Before the commencement of the actual data collection, a pretest was performed on 10% of the computed sample in the Birbir district, which has similar sociocultural and living standards as the study area. Necessary feedback and adjustment of the phrasing were performed accordingly. Internal Consistency was checked using Cronbach’s alpha and the result was above 0.7 for all constructs. Training was provided to all data collectors, and the supervisor performed a close follow-up and a random spot-check. The supervisor checked the questionnaire for consistency and completeness daily during data collection. The data collectors were then given feedback.

### Variables

#### Dependent variables

Intention to use preconception care.

#### Independent variables

Sociodemographic characteristics: Age, religion, educational status, occupational status, and marital status.

TPB constructs: Direct Attitude, direct subjective norm, direct perceived behavioral control, indirect attitude, indirect subjective norm, and indirect perceived behavioral control.

### Measurement and scoring

Intention to use preconception care was measured using five items on a five-point Likert scale response format (1 = strongly disagree to 5 = strongly agree). A composite score was obtained by summing all the items, and the score ranged from five to twenty-five. A higher score indicated a high intention to incline for preconception care (five items of a five-point Likert scale) [18].

Direct attitude was measured by five semantic differential items using a five-point scale. The composite score ranged from five to twenty-five, and a higher score indicated a positive attitude toward preconception care use.

Direct subjective norms were measured by five items on a five-point Likert scale. The five items were summed to form a direct subjective norm score, and the composite score ranged from five to twenty-five. A higher score indicated a higher social influence on using preconception care services.

Direct perceived behavioral control was measured by four items with a five-point Likert scale response format. The composite score ranged from four to twenty, and a higher score indicated higher perceived ability or less difficulty in using preconception care services.

Knowledge of preconception care was measured by 12 items based on correct responses scored out of 24 points. The mean score was calculated for analysis [19].

## Results

### Socio-demographic characteristics

A total of 415 women participated in the study, giving a response rate of 98.3%. The mean age of the participants was 28.4 (SD 5.18), with the majority of 272 (65.5%) falling between the ages of 25 and 34. More than half of the participants, 240 (57.8%), were protestants, while 145 (34.9%) were Orthodox Christians. In terms of marital status, 375 (90%) participants were married. Regarding educational and occupational status, 145 (35.7%) had completed secondary school, and 142 (34.2%) were housewives, respectively (Table 1).

**Table 1:** Socio-demographic characteristics of respondents in Arba Minch town, Southern Ethiopia,2022

Variables	Categories	Frequency	Percent (%)
Age	15–24	90	21.7
	25–34	272	65.5
	35–47	53	12.8
Religion	Orthodox	145	34.9
	Protestant	240	57.8
	Muslims	21	5.1
	Others*	9	2.2
Marital status	Married	375	90.4
	Single	20	4.8
	Divorced	16	3.8
	Widowed	4	1
Educational status	No formal education	39	9.4
	Primary school	139	33.5
	Secondary school	145	34.9
	Diploma and above	92	22.2
Occupation	Housewife	142	34.2
	Employed	101	24.4
	Merchant	78	18.8
	Student	42	10.1
	Daily Laborer	52	12.5

Notes: * Catholic = 4, Adventist = 3, Jehova = 2

### Obstetrics and reproductive health characteristics

In this study, 388 (93.5%) of the women had been pregnant before. Two hundred seventy-five (70.9%) of them were multigravida, 261 (67.3%) were multiparous, and 113 (30.1%) were primiparous. The majority (87.9%) of the respondents had at least one ANC visit during their last pregnancy, 127 (32.7%) had previous PNC contact, 161 (41.5%) had a planned pregnancy, 40 (10.3%) had a previous adverse pregnancy outcome, and 127 (68.0%) reported a history of using family planning.

### Source of information and knowledge of preconception care

Among the 415 participants, 140 (33.7%) women had ever heard about preconception care before. Healthcare workers, including health extension workers, composed 127 (30.6%) of the primary sources of information for those who had ever heard about preconception care, followed by 60 (14.5%) from mass media (TV, radio, social media, etc.), 38 (9.2%) from friends, and 34 (8.4%) from family members.

In terms of knowledge about ‘what should be done before pregnancy?’, 157 (37.8%) of the participants mentioned visiting a health facility, followed by 152 (36.6%) who mentioned family planning. Whereas weight should be maintained 12 (2.9%) and diet modification 8 (1.9%) were mentioned. For each item, scores were summed, and mean scores were computed. Accordingly, the mean score of knowledge was 12 (SD 6.6).

### Theory of planned behavior variables

The mean score of participants’ intention to use preconception care was 21.4 (SD 2.5). The mean score of participants’ direct attitude toward preconception care was 18.8 (SD 4.8), followed by 16.5 (SD 4.7) mean score of direct subjective norm about preconception care. For the indirect constructs, attitude had the highest mean of 107.7 (SD 24.5) (Table 2).

**Table 2:** Descriptive statistics of the theory of planned behavior variables and intention among reproductive-age women in Arba Minch Town, Southern, Ethiopia, 2022 (*N* = 415)

Variable	No of Items	Min. Value	Max. Value	Mean	SD	Cronbach’s alpha
Intention	5	5	25	21.4	2.5	0.70
Direct Attitude	5	5	25	28.8	4.8	0.93
Direct Subjective norm	5	5	25	16.5	4.7	0.85
Direct PBC	4	4	20	12.4	4.5	0.85
Indirect Attitude	12	6	150	107.7	24.5	0.83
Indirect Subjective norm	10	5	125	65.5	26.2	0.88
Indirect PBC	14	7	175	80.4	42.0	0.91

SD = standard deviation, PBC = perceived behavioral control, α = Cronbach’s alpha, Min and Max = minimum and maximum values

### Correlation of theory of planned behavior variables and other variables with intention

Karl Pearson’s correlation coefficients show that all of the theories of planned behavior variables had a significant correlation with intention at a *p*-value of < 0.01. Direct perceived behavioral control had the highest correlation, followed by indirect attitude and indirect subjective norm. Indirect perceived behavioral control had a negative correlation with intention (Table 3).

**Table 3:** Pearson correlation of theory of planned behavior variables among reproductive-age women of Arba Minch town, Southern, Ethiopia, 2022 (*N* = 415)

Variables	Intention	DAT	DSN	DPBC	IAT	ISN	IPBC	knowledge
Intention	1							
DAT	0.431**	1						
DSN	0.421**	0.607**	1					
DPBC	0.463**	0.530**	0.588**	1				
IAT	0.441**	0.769**	0.547**	0.446**	1			
ISN	0.441**	0.578**	0.745**	0.492**	0.549**	1		
IPBC	-0.363**	-0.552**	-0.525**	-0.661**	-0.428**	-0.439**	1	
Knowledge	0.259**	0.481**	0.444**	0.480**	0.412**	0.377**	-0.500**	1

Abbreviations: DAT: Direct Attitude: DSN: Direct Subjective Norms: DPBC: Direct Perceived Behavioral Control: IAT: Indirect Attitude: ISN: Indirect subjective norm: IPBC: Indirect perceived behavioral control

Note: ** The correlation is significant at the 0.01 level (two-tailed)

### Multiple linear regression

Level of education, knowledge, and all direct theory of planned behavior variables had a *p*-value of less than 0.025 in simple linear regression and age was included which was significant in the previous study. In multiple linear regression, age, attitudes (β = 0.201, *P* < 0.001, 95% CI = 0.088, 0.285), subjective norms (β = 0.158, *P* < 0.006, 95% CI = 0.045, 0.272), and perceived behavioral control (β = 0.263, *P* < 0.001, 95% CI = 0.152, 0.374) significantly predicted the intention to use preconception care. This suggests that a unit-positive change in women’s perception of their ability to influence the circumstances that prevent them from utilizing preconception care services will raise intention to use preconception care by 0.263 units, assuming that all other factors remain constant. Furthermore, a unit-positive change in women’s attitudes toward the benefits of preconception care will increase the intention to utilize preconception care services by 0.201 units when other characteristics remain constant. Additionally, women who believe their significant others will approve of using preconception care will increase their intention by 0.158 units, provided that all other factors are constant. The constructs of the model explained 27.0% (Adj. R^2^) of the variation in intention to use preconception care (Table 4).

**Table 4:** Multiple linear regression of intention to use preconception care and its predictors among reproductive-age women of Arba Minch town, Southern, Ethiopia, 2022 (*N* = 415)

Variables	Value	Standardized β	*P* Value	95% for CI β
Age	15–24(Ref)			
	25–34	-0.115*	0.023	-0.213, -0.016
	35–49	-0.087	0.091	-0.189, 0.014
Educational status	No formal education (Ref)			
	Primary school	0.106	0.156	-0.041,0.253
	Secondary school	0.090	0.245	-0.062,0.242
	Diploma and above	0.103	0.157	-0.040,0.247
Knowledge		-0.021	0.684	-0.124,0.081
Direct PBC		0.263*	0.000	0.152,0.374
Direct Attitude		0.201*	0.001	0.088,0.285
Direct SN		0.158*	0.006	0.045,0.272

*p < 0.05, Ref = References

## Discussion

This study assessed reproductive-age women’s intention to use preconception care and its predictors using the theory of planned behavior.

The current study found that the mean value of intention to use preconception care was 21.4 (SD 2.5). This finding is consistent with previous studies conducted in Malaysia [20] and France [21], in which the majority of women revealed an interest in preconception health care.

This study revealed that perceived behavioral control was the predominant and most influential predictor of the intended use of preconception care services. This finding was consistent with a study conducted in Southwest Ethiopia [22], southern Ethiopia [23], and Jimma, Ethiopia [24]. Ajzen’s theoretical assumptions [16] support this finding, suggesting that the greater women’s control over factors enabling or hindering their use of preconception care, the stronger their intention to utilize these services. However, a study conducted in Iran found that perceived behavioral control did not predict women’s intention to use pre-pregnancy care [17]. A similar outcome was reported in a study in the Netherlands [18], which examined constructs akin to perceived behavioral control, such as self-efficacy. These discrepancies could stem from variations in population characteristics, awareness levels regarding the behavior, and the availability of preconception services in the respective study areas.

In this study, attitude toward preconception care was ranked second in predicting the intention to use preconception care. These findings are consistent with studies conducted in Iran [25], Tanzania [26], southwest Ethiopia [22], and the Netherlands [18], where a positive attitude was identified as a significant predictor of intention to use preconception care services. This suggests that participants who perceive preconception care as beneficial are more likely to express intention to utilize these services compared to their counterparts.

According to the findings of this study, subjective norms were identified as another important predictor of intention to use preconception care. The finding is supported by Studies conducted in southwest Ethiopia [22], China [27], Nairobi (25), Norway (26), and Laresta, Iran 2016 (16). This implies that the individual’s intention to use preconception care is more likely to be influenced by important referent individuals such as families, friends, spouses, healthcare professionals, and neighbors. Therefore, interventions aimed at improving preconception care utilization may benefit from targeting these influential individuals collectively, rather than focusing solely on women within a specific age group. This approach is supported by Ajzen’s TPB assumptions [16], which suggest that the intention to engage in a behavior is determined by attitude, subjective norms, and perceived behavioral control.

The correlation analysis of this study showed that there is a direct relationship between the indirect and direct measurement items of the theory of planned behavior. This shows that the commonly held salient beliefs identified from the reviewed literature regarding attitude, subjective norms, and perceived behavioral control about preconception care were well explored through indirect constructs of each measurement. This indicates that indirect measures directly influence the direct measures of attitude, subjective norm, perceived behavioral control, and intention toward preconception care use, which is consistent with the principles of TPB [16].

The study’s findings have significant implications for preconception care policy and initiatives. The findings imply that strategies should prioritize increasing women’s control over healthcare access, promoting favorable attitudes through public health campaigns, and utilizing the impact of key social referents. Programs should focus on improving healthcare infrastructure, implementing education and awareness campaigns, involving communities, and incorporating preconception care into larger health programs. These tailored interventions can have the potential to improve preconception care uptake and mother-and-child health outcomes.

### Limitations of the study

Despite using an interviewer-administered questionnaire, there was a possibility of participants providing responses they perceived as socially desirable, potentially introducing social desirability bias.

As the nature of the study design was cross-sectional, it does not reflect cause-and-effect relationships. Furthermore, we cannot be certain that women who stated they intended to use preconception care services in the future intended to do so. The theory of planned behavior assumes that behavior is a linear decision and does not consider that it can change over time.

## Conclusion

The mean score of participants’ intention to use preconception care was 21.43 (SD 2.47), approaching the maximum possible score. This indicates a strong intention among participants to use preconception care. Perceived behavioral control emerged as the strongest predictor, followed by attitude and subjective norms, independently influencing the intention to use preconception care. These factors should be carefully considered by health professionals when designing interventions aimed at promoting preconception care among reproductive-age women.

## Data Availability

The datasets used and/or analyzed during the current study are available from the corresponding author upon reasonable request.

## Abbreviations

AT: Attitude
PBC: Perceived Behavioral Control
PCC: Preconception Care
SN: Subjective Norm
TPB: Theory of Planned Behavior
WHO: World Health Organization
